# Health-related quality of life of Malaysian patients with chronic non-malignant pain and its associated factors: a cross-sectional study

**DOI:** 10.1186/s12891-022-05354-1

**Published:** 2022-04-28

**Authors:** Ju-Ying Ang, E-Li Leong, Huan-Keat Chan, Asrul Akmal Shafie, Shi-Qi Lee, Punita Mutiah, Ronald Vei-Meng Lim, Chia-Ming Loo, R. Usha S. Rajah, Mazlila Meor Ahmad Shah, Zubaidah Jamil Osman, Lee-Choo Yeoh, Devanandhini Krisnan, Kavita Bhojwani

**Affiliations:** 1 Clinical Research Centre (CRC), Hospital Raja Permaisuri Bainun, Ministry of Health, Level 4, Ambulatory Care Centre (ACC), Jalan Raja Ashman Shah, 30450 Ipoh, Perak Malaysia; 2 Clinical Research Centre (CRC), Hospital Sultanah Bahiyah, Ministry of Health, Km6, 256, 05460 Alor Setar, Kedah Malaysia; 3 School of Pharmaceutical Sciences, Universiti Sains Malaysia, 11800 Gelugor, Penang, Malaysia; 4 Pharmacy Department, Hospital Pulau Pinang, Ministry of Health, Jalan Residensi, 10990 George Town, Pulau Pinang Malaysia; 5 Department of Anaesthesiology and Intensive Care, Hospital Pulau Pinang, Ministry of Health, Jalan Residensi, 10990 George Town, Pulau Pinang Malaysia; 6 Department of Anaesthesiology and Intensive Care, Hospital Selayang, Ministry of Health, Selayang - Kepong Hwy, 68100 Batu Caves, Selangor Malaysia; 7 Department of Anaesthesiology and Intensive Care, Hospital Sultanah Bahiyah, Ministry of Health, Km6, 256, 05460 Alor Setar, Kedah Malaysia; 8 Pain management Unit,Department of Anesthesiology and Intensive Care, Hospital Selayang, Ministry of Health, Selayang - Kepong Hwy, 68100 Batu Caves, Selangor Malaysia; 9 Management Science University, University Drive, Off Persiaran Olahraga, 40100 Shah Alam, Selangor Malaysia; 10 Pain Management Clinic, Department of Anaesthesiology and Intensive Care, Hospital Raja Permaisuri Bainun, Ministry of Health, Jalan Raja Ashman Shah, 30450 Ipoh, Perak Malaysia; 11 Department of Anaesthesiology and Intensive Care, Hospital Raja Permaisuri Bainun, Ministry of Health, Jalan Raja Ashman Shah, 30450 Ipoh, Perak Malaysia

**Keywords:** Quality of life, Chronic pain, Malaysia

## Abstract

**Background:**

Chronic pain has a major impact on a patient’s quality of life, affecting physical and psychological functioning. It has debilitating consequences on social and economic aspects too. This study aimed to explore the status of health-related quality of life (HRQoL) of Malaysian patients suffering from chronic non-malignant pain.

**Methods:**

Four hospitals offering pain clinic services were involved in this multicentre cross-sectional study conducted between June and September 2020. Adult patients who had been diagnosed with non-malignant chronic pain lasting for at least three months and able to communicate in English or Malay language were recruited in this study. Participants were informed about the study and were made aware that their participation was entirely voluntary. A battery of questionnaires consists of the EuroQol-5 dimensions-5 levels questionnaire (EQ-5D-5L) and the EuroQol visual analogue scale (EQ VAS), the Pain Self-Efficacy questionnaire (PSEQ) and the Pain Catastrophizing Scale (PCS) were self-administered by the patients. Besides, a structured questionnaire was used to collect their socio-demographic information, pain condition, sleep quality and working status. Participants’ usage of pain medications was quantified using the Quantitative Analgesic Questionnaire (QAQ).

**Results:**

A total of 255 patients participated in this study. A median EQ-5D index value of 0.669 (IQR: 0.475, 0.799) and a median EQ VAS score of 60.0 (IQR: 50.0, 80.0) were recorded. Malay ethnicity (Adj. B: 0.77; 95% CI: 0.029, 0.126; *p* = 0.002) and a higher level of self-efficacy (Adj. B: 0.008; 95% CI: 0.006, 0.011; *p* < 0.001) were predictors of a better HRQoL, while suffering from pain in the back and lower limb region (Adj. B: -0.089; 95% CI: − 0.142, − 0.036; *p* = 0.001), the use of a larger amount of pain medications (Adj. B: -0.013; 95% CI: − 0.019, − 0.006; *p* < 0.001), and a higher degree of pain magnification (Adj. B: -0.015; 95% CI: − 0.023, − 0.008; *p* < 0.001) were associated with a poorer HRQoL.

**Conclusions:**

These findings suggested that Malay ethnicity and a higher level of self-efficacy were predictors of a better HRQoL in patients with chronic pain, whereas pain-related factors such as higher usage of medication, specific pain site and pain magnification style were predictors of poorer HRQoL.

## Background

Pain is defined as an “unpleasant sensory and emotional experience associated with, or resembling that associated with, actual or potential tissue damage” [1]. It can be classified based on either its aetiology (i.e., nociceptive, neuropathic, or mixed pain) or duration (i.e., acute or chronic pain). According to the International Association for the Study of Pain, pain is only considered chronic if it lasts for at least 3 months [2, 3]. In Asia, the prevalence of chronic pain widely ranges from 7.1% in Malaysia to 34.9% in Hong Kong [4, 5]. Chronic pain becomes more common as people age, with 15.2% of Malaysia’s elderly suffering from it [5].

Chronic pain has various clinical, social and economic consequences. In the UK, the management of chronic pain incurs a cost of £69 million annually [6]. Similarly, approximately ¥5 billion was spent on the management of low back pain between 1995 and 1997 in Japan [7]. In Malaysia, patients with chronic pain are highly dependent on the public healthcare facilities for care [5], and it is clear that the financial burden falls mainly on the public healthcare system.

Several studies reported that patients with chronic pain had a similar health-related quality of life (HRQoL) to those with end-stage cancer [8] but a lower HRQoL than those with stroke [9]. Frequent and intense pain [10], as well as chronic pain with neuropathic characteristics, have been shown to reduce their HRQoL [11]. Apart from persistent discomfort, the multiple physical and psychological changes associated with chronic pain have greatly affected the lives of patients, mainly by causing depression, sleep disturbance, changes in their personality and social relationships [6, 8, 12], absenteeism and productivity loss [13]. Thus, chronic pain is a medical condition that has a significant impact on a patient’s quality of life, both in physical and psychological functioning.

The HRQoL could be measured by using either a disease-specific or a generic approach. Although the disease-specific approach (e.g., Brief Pain Inventory) captures the symptoms or functions of a patient [14], it does not allow comparisons across different medical conditions. Therefore, the generic approach has become more popular in recent years, and some instruments developed for this purpose even provide health state utility values for cost-utility analyses [11, 14], which enable more effective decision making and resource allocation. In Malaysia, studies on chronic pain mainly focused on the effectiveness of different treatment modalities and procedures, while the HRQoL of patients with chronic pain is yet to be explored. As pain is perceived differently across cultures and regions [15], this study aimed to explore the HRQoL of Malaysian patients suffering from chronic non-malignant pain using a generic approach.

## Methods

A multicentre cross-sectional study was undertaken between June and September 2020 in four hospital-based pain clinics, which provide clinical service to approximately more than 400 patients with non-malignant chronic pain altogether monthly. The study was approved by the Medical Research & Ethics Committee (NMRR-20-558-53,144 (IIR), Ref: KKM/NIHSEC/P20–754(5)). Adults (≥18 years of age) who had non-malignant pain lasting for at least 3 months and being seen in the pain clinic during the data collection period were included in the study, whereas those who were unable to communicate in English or Malay language were excluded. All eligible patients were approached to participate during the study period.

### Data collection

Written consent was obtained from all the study participants. A structured questionnaire was used to collect self-reported socio-demographic information, pain intensity (minimum, usual, and maximum pain scores) at the time of the study and within 1 month before the study, as well as sleep quality and working status within 1 month of the study. Additionally, participants were required to complete four validated questionnaires. The first two questionnaires were the EuroQol-5 dimensions-5 levels (EQ-5D-5L) and the EuroQol visual analogue scale (EQ VAS), which measured their HRQoL [16]. In the EQ-5D-5L, the participants rated their physical and mental condition on the day of study using a 5-level scale, from which an EQ-5D index value was generated. They also indicated their perceived health status on a VAS ranging from 0 to 100 in the EQ VAS. The Pain Self-efficacy Questionnaire (PSEQ) was used to measure the degree of self-efficacy in daily functioning while coping with chronic pain [17]. The Pain Catastrophizing Scale (PCS) was used to assess the degree of catastrophizing in three aspects, namely rumination, helplessness and magnification of pain [18]. The scoring system, outcome measurement and psychometric properties of all the four instruments are summarized in Table 1. The participants were allowed to choose either English or Malay versions of each instrument in responding to the questionnaires.

**Table 1:** Four validated questionnaires used in this study and their psychometric properties

Instrument	Total number of items/ domains covered	Scoring	Outcome measurement	Psychometric properties i English version ii Malay version
1.1. EQ-5D-5 L [16]	5 items cover 5 dimensions: • Mobility • Self-care • Usual activities • Pain/discomfort • Anxiety/depression	Each dimension is scored based on a 5-level scale, ranging from “no problem” (level 1) to “unable to perform/ having extreme problem” (level 5)	**EQ-5D-5L health state:** A state of ‘11111’ represents no problem in all domains, whereas ‘55555’ represents an extreme problem in all domains.	**Kappa agreement** [19]: i. 0.208 (self-care) to 0.382 (anxiety/depression) ii. -0.015 (self-care) to 0.553 (mobility)
			**EQ-5D index value** [20]**:** The Malaysian utility value set was used in this study, with a possible value ranging from −0.442 to 1. A value of ‘1’ represents the state of full health, ‘0’ represents a health state equivalent to dead, and ‘<0’ represents a health state worse than dead.	
2. EQ VAS [16]	1 item indicates the perceived health status	A Visual Analogue Scale ranging from 0 to 100	**EQ VAS score:** A score of ‘0’ indicates the worst imaginable health, and ‘100’ indicates the best imaginable health.	**Pearson correlation coefficients** [21]**:** i & ii. EQ VAS and Mental component score-12 item = 0.2 i & ii. EQ VAS and Physical component score-12 item = 0.4
3. PSEQ [17]	10 items evaluate self-efficacy in performing activities despite pain	Each item is scored on a 7-point scale, ranging from “0 = not at all confident” to “6 = completely confident”.	**PSEQ score:** The possible score ranges from 0 to 60; a higher score indicates greater confidence in dealing with pain	**Cronbach’s alpha:** i 0.92 [17] ii 0.95 [22]
4. PCS [18]	13 items cover 3 domains: • rumination (sum of items 8–11) • helplessness (sum of items 1–5, 12) • magnification (sum of items 6, 7, 13)	Each item is scored on a 5-point Likert scale, ranging from “0 = not at all” to “4 = all the time”.	**PCS sum score:**The summation of all 13 items, with a possible score ranging from 0 to 52; a higher score indicates a higher pain catastrophizing tendency.	**Cronbach’s alpha:** i 0.87 [18] ii 0.90 [23]
			**PCS subscale score:**The summation of items under the respective domain, with the possible subscale score ranges as follows: Rumination: 16, Helplessness: 24, Magnification: 12; a higher subscale score represents a greater level of rumination, helplessness or magnification.	

The medical history of each participant was extracted from their medical records using a standardized data collection sheet. Information collected included their clinical diagnosis, as well as both pharmacological and non-pharmacological treatments received. The medications used to manage the pain within 1 month before the study were also recorded, including opioids, nonsteroidal anti-inflammatory drugs (NSAIDs), anticonvulsants, selective serotonin and norepinephrine reuptake inhibitors (SSRIs and SNRIs), tricyclic antidepressants (TCAs), muscle relaxants and anticholinergic drugs. The use of these pain medications was subsequently quantified based on the Quantitative Analgesic Questionnaire (QAQ) [24], in which a higher QAQ score represented a larger amount of medication used. The maximum dose of each medication was set based on the Malaysian Pain Management Handbook, the Malaysian Low Back Pain Management Guideline and the Malaysian Drug Formulary [25–27].

### Data analysis

The descriptive analysis of categorical variables was summarized as frequencies and percentages. Numerical variables were summarized as means and standard deviations (SDs), or as medians with interquartile ranges (IQRs). The primary endpoint of this study was the HRQoL of the participants which was expressed as an EQ-5D index value, calculated based on the Malaysian value set [20]. Furthermore, the predictors of HRQoL were identified using the stepwise multiple linear regression analysis, with the *p*-value set at 0.05. Interaction terms were checked for the final model. All statistical analysis was conducted using the SPSS version 20.0 (IBM, New York).

## Results

A total of 360 patients with non-malignant chronic pain were approached and 255 of whom participated in the study, yielding a response rate of 70.8%. The patients excluded were those who declined to participate (*n* = 40), were unable to communicate in English or Malay (*n* = 57), had visual impairment (*n* = 1), had an acute exacerbation of pain or felt unwell at the time when they were approached (*n* = 7). Only 246 patients completed the survey and were included in the final analysis. Approximately half of them were female (61.4%), of Malay ethnicity (50.8%) and had a secondary education level (58.2%) (Table 2). Approximately 40% either lost their job (21.1%) or had their job scope adjusted (22.4%) due to pain, while some were on sick leave frequently (13.4%).

On average, the participants experienced pain for 6 (IQR: 2, 12) years, had been followed up at one of the pain clinics for 2.5 years (IQR: 0.8, 6.2), and had 1 (IQR: 0, 2) comorbidity. Most of them had one pain site (69.1%), and only one had four pain sites. The most common pain sites were the back, sacrum, buttock and the lower limb region (71.1%), and 76.4% of them suffered from neuropathic pain. A total of 35 patients had at least one psychiatric disorder, with depressive disorder (11.4%) as the most common diagnosis. In the 1 month before this study, more than half (69.8%) of the participants experienced continuous pain, with a median minimum pain score of 3 (IQR: 2, 5) and a median maximum pain score of 8 (IQR: 7, 9). Less than 10% did not take any medications to relieve the pain (QAQ score = 0), more than half took their medication as prescribed (65.9%), while 24.8% reduced the dose or frequency of their medications. The characteristics and health conditions of participants are summarized in Table 2.

**Table 2:** Characteristics and health conditions of participants in the one month before the day of study (*n* = 246)

**Age, year, mean (SD**^**a**^**)**	52.8	(14.2)
**Gender**, n (%)		
Male	95	(38.6)
Female	151	(61.4)
**Ethnicity**, n (%)		
Malay	125	(50.8)
Non-Malay	121	(49.2)
**Education level**^b^, n (%)		
Tertiary	90	(36.9)
Secondary	142	(58.2)
Primary or no formal schooling	12	(4.9)
**Marital status**^b^, n (%)		
Married	185	(75.8)
Single	37	(15.2)
Divorced or widowed	22	(9.0)
**Working status**^c^, n (%)		
Unemployed or not working due to pain	52	(21.1)
Sick leave frequently required due to pain	33	(13.4)
Having job adjusted due to pain	55	(22.4)
Job not affected by pain	31	(12.6)
Not working even before pain	85	(34.6)
**Pain site**^c^, n (%)		
Head, face, mouth	33	(13.4)
Neck and upper limb	94	(38.2)
Back/sacrum/buttock and lower limb	175	(71.1)
Abdomen, pelvis, chest	29	(11.8)
**Neuropathic pain**, n (%)	188	(76.4)
**Psychiatric diagnosis**^c^, n (%)		
Depressive disorder	28	(11.4)
Anxiety disorder	6	(2.4)
Others^d^	7	(2.8)
**Pain score**^e^, median (IQR^f^)		
Minimum^b^	3	(2, 5)
Usual^g^	5	(4, 6)
Maximum	8	(7, 9)
**Sleep quality**^e,g^, n (%)		
Good	50	(20.4)
Acceptable	102	(41.6)
Poor	93	(38.0)
**Self-adjustment of medication**, n (%)		
Used medications as prescribed	162	(65.9)
Increased dose/frequency	20	(8.1)
Reduced dose/frequency	61	(24.8)
Increased & decreased dose/frequency	3	(1.2)
**Quantitative Analgesic Questionnaire score** (QAQ)^e^, median (IQR^f^)	4	(2, 7)
**Pain Self-Efficacy Questionnaire** (PSEQ) score, mean (SD^a^)	34.3	(12.8)
**Pain Catastrophizing Scale** (PCS) sum score, mean (SD^a^)	26.9	(12.8)
Subscore: Rumination, mean (SD^a^)	9.5	(4.2)
Subscore: Magnification, mean (SD^a^)	5.7	(3.5)
Subscore: Helplessness, mean (SD^a^)	11.7	(6.2)

^a^*SD* Standard deviation

^b^*n* = 244 due to two incomplete data

^c^More than one option was allowed

^d^Including schizophrenia, bipolar disorder, adjustment disorder, insomnia, alcohol use disorder

^e^In the one month before the study

^f^IQR interquartile range

^g^*n* = 245 due to one incomplete data

**Fig. 1:**
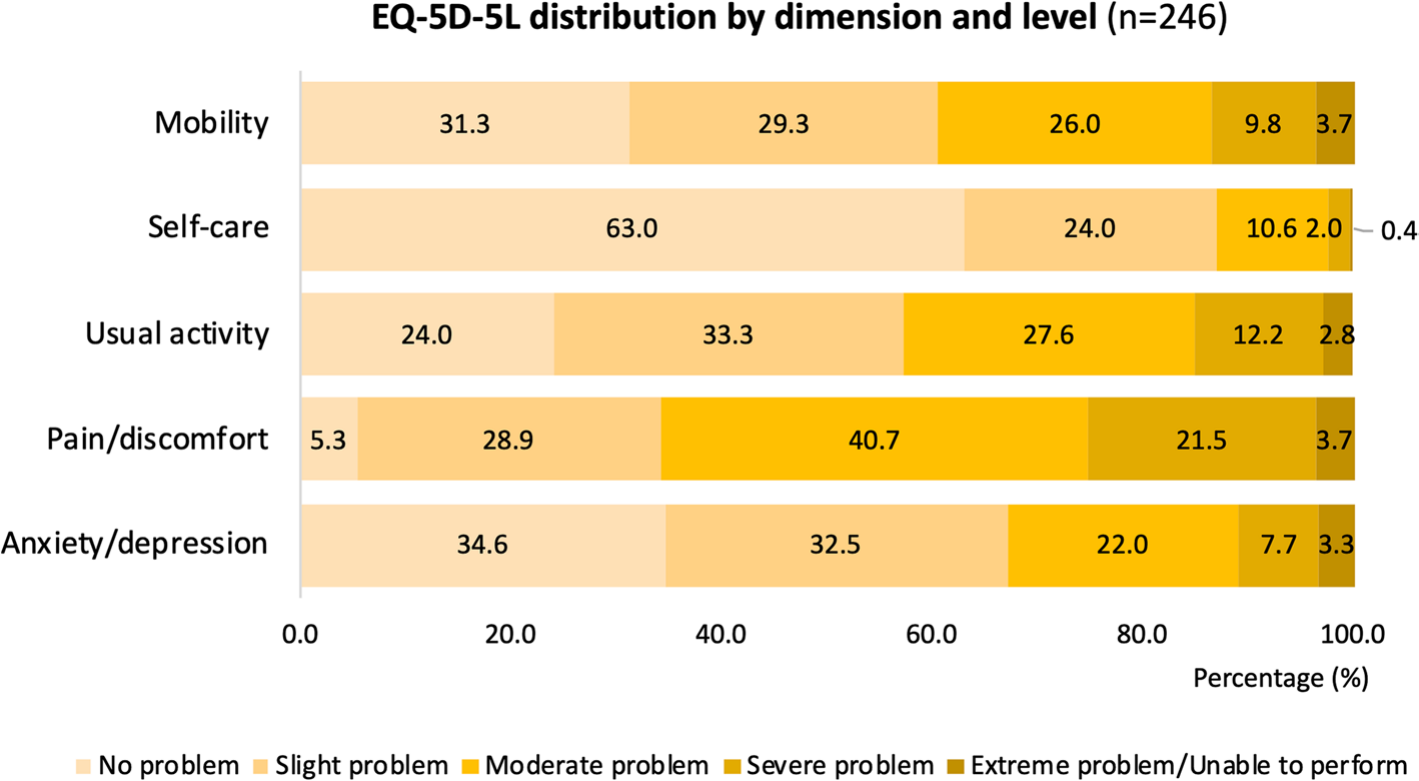
The distribution of EQ-5D-5L responses among participants by dimension and level

The patients reported a median pain score of 5 (IQR: 4, 7) on the day of the study, and only 4.1% of them reported a health state of 11,111 in the EQ-5D-5L questionnaire, which represented having no problem in any of the five dimensions tested. More than half of them had no difficulty with self-care (63.0%). However, 65.9% of patients reported having a moderate to a severe problem with pain/discomfort, followed by usual activity (42.6%), mobility (39.5%) and anxiety/depression (33.0%) (Fig. 1). A median EQ-5D index value of 0.669 (IQR: 0.475, 0.799) and a median EQ VAS score of 60.0 (IQR: 50.0, 80.0) were recorded.

Predictors of HRQoL included ethnicity, pain site, QAQ score, PSEQ sum score and the magnification subscore of PCS (Table 3). Participants of Malay ethnicity recorded a better HRQoL (Adj. B: 0.77; 95% CI: 0.029, 0.126; *p* = 0.002). Additionally, a better HRQoL was recorded in the participants with greater self-efficacy represented by the PSEQ sum score (Adj. B: 0.008; 95% CI: 0.006, 0.011; *p* < 0.001). On the contrary, a poorer HRQoL was more likely to occur among those who suffered from pain in the back, sacrum, buttock or lower limb region, as opposed to those who did not experience pain in these regions. (Adj. B: -0.089; 95% CI: − 0.142, − 0.036; *p* = 0.001). Those who had a higher level of pain magnification, as indicated by the PCS sub-scale score of magnification, were more likely to have a poorer HRQoL (Adj. B: -0.015; 95% CI: − 0.023, − 0.008; *p* < 0.001). Also, patients who used more pain medications, as indicated by a higher QAQ score, were more likely to have a poorer HRQoL (Adj. B: -0.013; 95% CI: − 0.019, − 0.006; *p* < 0.001).

**Table 3:** Predictors of the EQ-5D-5L index value among patients with chronic pain (*n* = 246)

Predictors	Multiple Linear Regression		
	Adj. B^d^	(95% CI^e^)	*p-*value
Malay ethnicity	0.077	(0.029, 0.126)	0.002
Pain site: back, sacrum, buttock or the lower limb region	-0.089	(−0.142, −0.036)	0.001
QAQ^a^ Score	-0.013	(−0.019, −0.006)	< 0.001
PSEQ^b^ Sum score	0.008	(0.006, 0.011)	< 0.001
PCS^c^ subscore: Magnification	-0.015	(− 0.023, − 0.008)	< 0.001

^a^*QAQ* Quantitative Analgesic Questionnaire

^b^*PSEQ* Pain Self-Efficacy Questionnaire

^c^*PCS* Pain Catastrophizing Scale

^d^*Adj. B* Adjusted regression coefficient

^e^*CI* Confidence interval

## Discussion

This study quantified the HRQoL of Malaysian patients with chronic non-malignant pain and reported its corresponding health utility values and predictors. The median health utility value of the patients with chronic pain reported in this study (0.669) is lower than that of the general Malaysian population (median: 1.0; IQR: 0.925, 1.075) [21], suggesting that chronic pain negatively affects quality of life. Furthermore, it is noted that chronic pain affected HRQoL to a greater extent as compared with other diseases, such as diabetes mellitus, cardiovascular diseases, chronic obstructive pneumonia disease, human immunodeficiency virus infection, chronic kidney disease and neoplasm [28]. This could be due to the various consequences of chronic pain, which compromise not only one’s physical function but also social life [10]. This study provides insight into the impact of chronic pain on the quality of life of patients, calling for greater attention to the effectiveness of pain management.

Several predictors of patients’ HRQoL were also identified in this study. Malay patients are more likely to have better HRQoL than patients of other ethnic groups. Although ethnic variation in HRQoL has not been reported in previous studies from Malaysia [29–32], a similar trend was shown in the US and England [33, 34]. Such variations were associated with differences in socioeconomic and health status among ethnic groups [33, 34]. A study conducted in Singapore, which is socio-culturally similar to Malaysia, also found that ethnicity has an impact on the HRQoL in addition to socioeconomic status [35, 36]. This implies the role of cultural diversity in health belief and perception. Although cultural influences on pain experience and expression are documented in other countries [15, 37], such information is currently limited in Malaysia. It is thus important to explore cultural differences in perceptions of pain as well as HRQoL among Malaysian patients.

Apart from ethnicity, this study also revealed that individuals who experienced pain involving back to lower limb region have a poorer HRQoL. This is consistent with the previous studies which suggest that HRQoL among patients with low back and knee pain is generally poorer [38–40]. Pain in the back and lower limbs commonly limits one’s physical function and mobility [41, 42], and affects one’s capacity to work [43]. Given its negative effect on patients, various treatment modalities are recommended to manage chronic pain. Nevertheless, an early intervention on pain in the back or lower limbs is also important to minimize its chronic complications. Besides the pain site, poorer HRQoL was also found in patients who used a larger amount of pain medications. Therefore, it is important to consider the use of non-pharmacological methods [44] in the management of chronic pain, such as exercise and acupuncture, which could help to provide alternative to manage pain instead of only relying on the pain medications in coping with pain.

Previously, when pain catastrophizing was studied as a single construct, it had already been shown to reduce the HRQoL of patients [45–47]. This study shows that, from the three dimensions of pain catastrophizing (i.e., rumination, helplessness and magnification), pain magnification significantly reduced patients’ HRQoL. A patient who magnifies pain tends to exaggerate the threat associated with pain. Such behaviour has been observed to have an impact on the physical aspect of HRQoL, likely as a result of a greater perceived barrier to activity involvement [48]. Thus, psychological interventions are expected to play an important role in pain management, as they assist patients dispel negative thoughts and live a better life [22, 49]. On the other hand, patients with higher self-efficacy had better HRQoL, which was consistent with previous literature [50]. Self-efficacy has been associated with improved physical function, reduced affective distress and lower pain severity in patients with chronic pain [51, 52]. Therefore, it is essential to assist patients in enhancing their self-efficacy in order to improve the outcome of pain management.

### Strengths and limitations

To the best of our knowledge, this is the first study assessing and quantifying the HRQoL of Malaysian patients with chronic non-malignant pain. Findings could serve as the baseline for comparisons with other diseases, as well as for cost-utility analysis of interventions on chronic pain in Malaysia. However, the data collection in this study was based mainly on self-reporting, and recall bias is therefore possible.

## Conclusion

This study reported the HRQoL utility value of patients with chronic non-malignant pain. Malay ethnicity and a higher level of self-efficacy were predictors of a better HRQoL, but suffering from pain in the back and lower limb region, the use of a larger amount of pain medications, and a higher degree of pain magnification were associated with poorer HRQoL.

## Data Availability

All data generated or analysed during this study are included in this published article.

## Abbreviations

EQ VAS: EuroQol visual analogue scale
EQ-5D-5L: EuroQol-5 dimensions-5 levels
HRQoL: Health-related quality of life
IQR: Interquartile range
NSAID: Nonsteroidal anti-inflammatory drug
PCS: Pain catastrophizing scale
PSEQ: Pain self-efficacy questionnaire
QAQ: Quantitative analgesic questionnaire
SD: Standard deviation
